# Supplementary material for: Complexome profiling of the Chlamydomonas psb28 mutant reveals TEF5 as an early PSII assembly factor
Source: Plant Cell. 2025 Mar 17;37(6):koaf055. doi: 10.1093/plcell/koaf055 (PMC12164590; doi:10.1093/plcell/koaf055)
Supplement: koaf055_Supplementary_Data [file koaf055_supplementary_data.zip › psb28_interactive.zip!/plots/intensity/qualityPlots/peptides/FKB16-3_Cre12.g530300.t1.2.html]

### Cre12.g530300.t1.2

Trivial name: FKB16-3  
SeqCoverage [median]: 30.80  
Mass: 21.77 kDa  
  
 

| Sequence | MissedCleavage | Mass [Da] | wtIntensity | muIntensity | Score |
| --- | --- | --- | --- | --- | --- |
| GLDDGLLDMKPGGIR | 1 | 1555.80 | 682.59 | 63965.98 | 107.14 |
| GLDDGLLDMKPGGIRR | 2 | 1711.90 | 477.12 | 18742.75 | 94.55 |
| LESAPEVSLPSGLK | 0 | 1425.77 | 2166.68 | 57210.60 | 186.87 |
| LYIPGDMAFPK | 0 | 1250.64 | 0.00 | 38180.45 | 93.18 |
| RLYIPGDMAFPK | 1 | 1406.74 | 0.00 | 10213.51 | 65.56 |
| VFDSSLDK | 0 | 909.44 | 0.00 | 8460.35 | 87.32 |
| VFDSSLDKGKPYDIR | 2 | 1738.89 | 1334.64 | 11332.21 | 74.48 |
| VGAGQVIK | 0 | 770.47 | 12040.05 | 81525.57 | 187.14 |
|  |  |  | 16701.1 | 289631.4 |  |
